# Targeting the endothelin axis as a therapeutic strategy for oral cancer metastasis and pain

**DOI:** 10.1038/s41598-020-77642-6

**Published:** 2020-11-30

**Authors:** Dongmin Dang, Yi Ye, Bradley E. Aouizerat, Yogin K. Patel, Dan T. Viet, King Chong Chan, Kentaro Ono, Coleen Doan, Johnny D. Figueroa, Gary Yu, Chi T. Viet

**Affiliations:** 1 Department of Oral and Maxillofacial Surgery, New York University, New York, NY USA; 2 Bluestone Center for Clinical Research, New York University, New York, NY USA; 3 Rory Meyers College of Nursing, New York University, New York, NY USA; 4 Division of Oral and Maxillofacial Radiology, Section of Hospital Dentistry, Columbia University Irving Medical Center, New York, NY USA; 5 Department of Physiology, Kyushu Dental University, Kitakyushu, Japan; 6 Department of Oral and Maxillofacial Surgery, Loma Linda University School of Dentistry, Loma Linda, CA USA; 7 Department of Basic Sciences, Center for Health Disparities and Molecular Medicine, Loma Linda University School of Medicine, Loma Linda, CA USA

**Keywords:** Cancer, Cancer therapy, Head and neck cancer, Oral cancer, Pain, Epigenetics, DNA methylation, Gene silencing

## Abstract

Metastasis reduces survival in oral cancer patients and pain is their greatest complaint. We have shown previously that oral cancer metastasis and pain are controlled by the endothelin axis, which is a pathway comprised of the endothelin A and B receptors (ET_A_R and ET_B_R). In this study we focus on individual genes of the pathway, demonstrating that the endothelin axis genes are methylated and dysregulated in cancer tissue. Based on these findings in patients, we hypothesize that ET_A_R and ET_B_R play dichotomous roles in oral carcinogenesis and pain, such that ET_A_R activation and silenced ET_B_R expression result in increased carcinogenesis and pain. We test a treatment strategy that targets the dichotomous functions of the two receptors by inhibiting ET_A_R with macitentan, an ET_A_R antagonist approved for treatment of pulmonary hypertension, and re-expressing the ET_B_R gene with adenovirus transduction, and determine the treatment effect on cancer invasion (i.e., metastasis), proliferation and pain in vitro and in vivo. We demonstrate that combination treatment of macitentan and ET_B_R gene therapy inhibits invasion, but not proliferation, in cell culture and in a mouse model of tongue cancer. Furthermore, the treatment combination produces an antinociceptive effect through inhibition of endothelin-1 mediated neuronal activation, revealing the analgesic potential of macitentan. Our treatment approach targets a pathway shown to be dysregulated in oral cancer patients, using gene therapy and repurposing an available drug to effectively treat both oral cancer metastasis and pain in a preclinical model.

## Introduction

Despite continued progress in developing targeted therapy, oral squamous cell carcinoma (SCC) patients continue to suffer from poor survival and pain. Treatment failure results in recurrence or metastasis. Patients who fail treatment suffer from pain produced by the cancer. Oral SCC patients have significantly more pain than other cancer patients^1^. We and others have shown that metastasis correlates with increased pain and poor orofacial function^2,3^. In this study we propose that the clinical correlation between oral SCC proliferation, metastasis and pain are the consequence of a common molecular pathway that involves signaling between cancer cells and the surrounding nerves. We explore one such pathway named the endothelin axis. The endothelin axis consists of the following: (1) three isoforms of the endothelin peptide hormone (ET-1, ET-2 and ET-3), (2) two distinct G protein-coupled receptors (ET_A_R and ET_B_R), and (3) ET-converting enzymes (ECE-1 and ECE-2), which activate the pro-forms of the peptide. Previous work from our laboratory on the endothelin axis genes have demonstrated that *EDNRB,* the gene for ET_B_R, is silenced by methylation in oral SCC tissue from patients. Furthermore, *EDNRB* methylation correlates with neck metastasis in oral SCC patients. Re-expression of *EDNRB* on cancer cells produces antinociception via endogenous opioid secretion. Conversely, activation of ET_A_R by ET-1 produces oral cancer nociception, and ET_A_R antagonism inhibits nociception in a mouse oral SCC model^4,5^.

Our findings thus far in patient tissues and preclinical models suggest that the endothelin axis genes play an important role in oral SCC invasion and pain. However what remains unknown is the distinct contribution of each endothelin axis component to cancer neuron signaling, and ultimately, to cancer invasion, proliferation and pain. Furthermore, a treatment strategy targeting the multiple dysregulated components of the pathway at the same time, has yet to be developed. In this study we hypothesize that the cancer cell employs endothelin axis gene dysregulation, specifically ET_B_R gene silencing and ET_A_R gene overexpression, to mediate processes of invasion, proliferation, and pain. We first confirm dysregulation of these endothelin axis genes in oral SCC tissues from patients. Next, we develop a treatment strategy involving inhibition of ET_A_R with macitentan, an ET_A_R antagonist approved for treatment of pulmonary hypertension, and re-expression of the ET_B_R gene with adenoviral gene therapy. We determine the efficacy of this strategy in treating cancer invasion, proliferation, and pain using in vitro and preclinical models.

## Results

### Endothelin pathway genes are dysregulated in human oral SCC tissues

**Figure 1:**
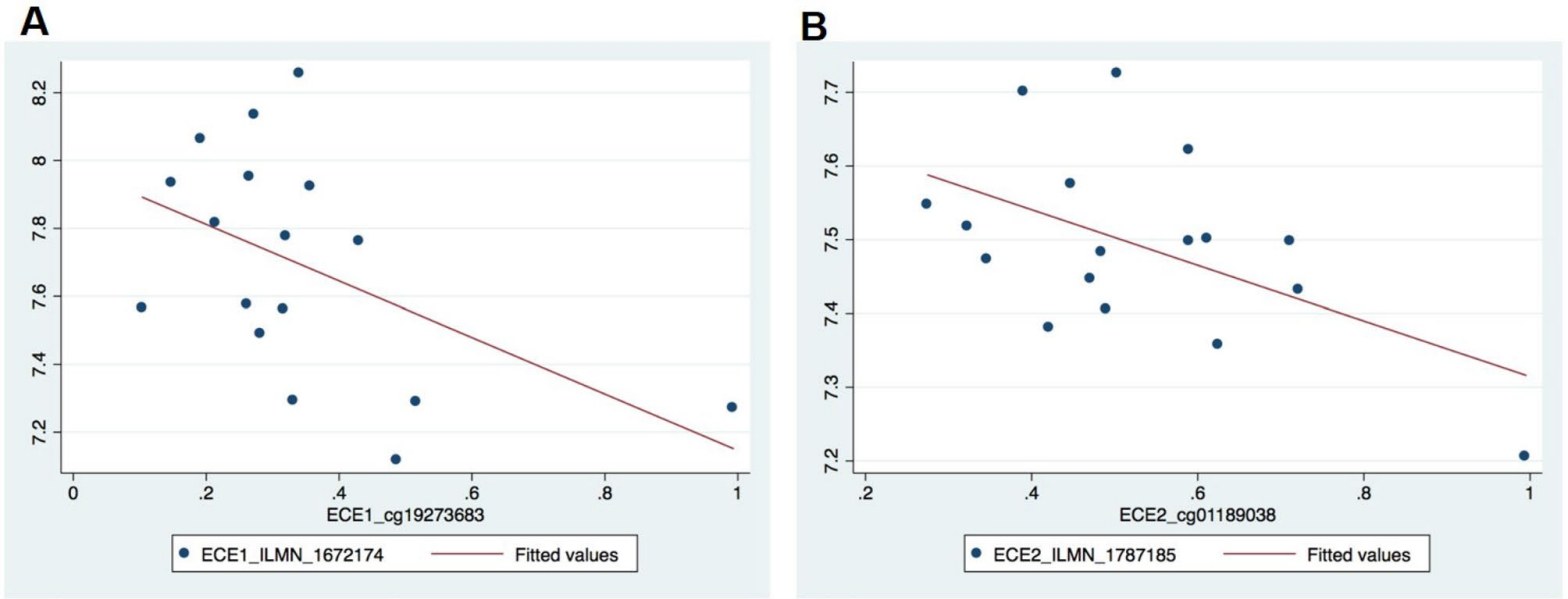
Linear regression models of *ECE1* and *ECE2* methylation and expression. The x-axis represents mean methylation; the y-axis represents mean expression level. There is an inverse relationship between (**A**) ECE1 and (**B**) ECE2 methylation at the analyzed CpG site and gene transcript level (*ECE1* r =  − 0.4985, *p* = 0.0417, *ECE2*; r = − 0.5290, *p* = 0.0290). For every 10% increase in CpG site methylation, the expression of ECE1 decreased by 0.0832 units (95% CI:  − 0.1629,  − 0.0036; *p* = 0.042). For every 10% increase in CpG site methylation, the expression of ECE2 decreased by 0.0377 units (95% CI:  − 0. 0710,  − 0.0044; *p* = 0.029).

Clinical relevance of a gene pathway involves demonstrating its dysregulation in human disease. Having established in a separate cohort of oral SCC patients that *EDNRB* is hypermethylated and silenced in cancer tissue, but not contralateral normal tissue^1^, we broadened our focus to determine the extent of dysregulation in the remaining endothelin axis genes. We quantified *ECE1, ECE2, EDN1, EDNRA,* and *EDNRB* methylation and gene expression with array data from a separate group of 22 oral SCC patients*.* Our significant findings were as follows: (1) there was an inverse relationship between EDNRA and EDNRB expression (*p* = 0.02, EDNRB_ILMN_1751904 vs. EDNRA_ILMN_1796629), and (2) the expression of *ECE1 and ECE2* were inversely correlated with methylation, i.e.*,* hypermethylated samples had lower gene expression (Fig. 1). *ECE1* CpG site cg19273683 was inversely correlated (r = −0.4985, *p* = 0.0417) with ECE1 mRNA levels. Using linear regression analysis, we found that for every 10% increase in CpG site methylation, the expression of ECE1 decreased by 0.0832 units (95% CI: − 0.1629, − 0.0036; *p* = 0.042). *ECE2* CpG methylation was inversely correlated with ECE2 transcript 1 levels (NM_014693.2, ECE2_ILMN_1787185; r = − 0.5290, *p* = 0.0290). For every 10% increase in CpG site methylation, the expression of ECE2 decreased by 0.0377 units (95% CI: − 0. 0710, − 0.0044; *p* = 0.029). Taken together, our findings in patient tissues suggested that EDNRA and EDRB expression were inversely related, and that *EDNRB, ECE1* and *ECE2* were dysregulated by methylation in oral SCC.

### Combination treatment of macitentan and *EDNRB* gene therapy inhibits oral cancer invasion in vitro

**Figure 2:**
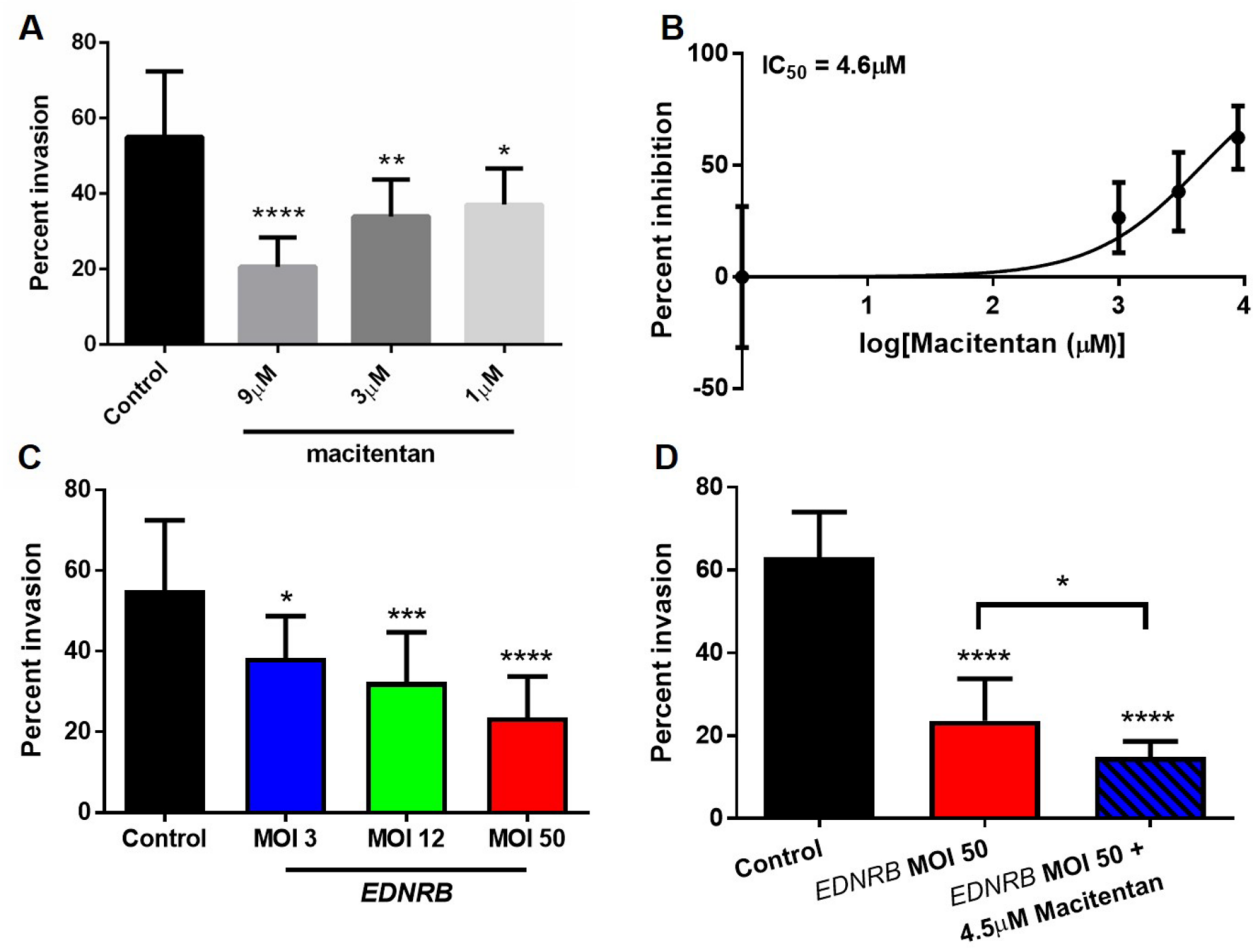
Macitentan inhibits Hela-O3 invasion in vitro. (**A**) Hela-O3 invasion is significantly reduced with macitentan treatment in a dose-dependent manner (one-way ANOVA, Holm Sidak test, *p < .05, **p < .01, ****p < .0001, compared to control). (**B**) A macitentan dose response curve based on doses used in Fig. 1A demonstrates that the IC50 value is 4.6 µM. (**C**) Ad-EDNRB virus transduction inhibits Hela-O3 invasion in a dose dependent manner (one-way ANOVA, Holm Sidak test, *p < .05, ***p < .001, ****p < .0001). (**D**) combination treatment of Ad-EDNRB and macitentan at IC50 dose is more effective in inhibiting Hela-O3 invasion than control (one-way ANOVA, Holm Sidak test, ****p < .0001) or Ad-EDNRB treatment alone (*p < .05).

The three outcome variables we measured in this study were invasion (i.e., metastasis), proliferation, and pain, with metastasis having the most significant effect on a patient’s overall survival^2^. We first determined whether our treatment strategy of inhibiting ET_A_R with macitentan and re-expressing *EDNRB* (the gene for ET_B_R) could effectively block cancer invasion in vitro and in a preclinical model of oral SCC. The in vitro invasion model was established with Hela-O3, a cell line with high invasive potential^3^. We showed that macitentan inhibited Hela-O3 invasion in a dose dependent manner compared to vehicle control (Fig. 2A). We calculated the IC50 value (dose required to inhibit invasion by 50%) by comparing the percent inhibition for the doses used in Fig. 2A. The IC50 for macitentan was 4.6 µM (Fig. 2B)*.*

We next determined whether *EDNRB* transduction alone inhibited Hela-O3 invasion, compared to control cells transduced with *GFP.* We determined that *EDNRB* gene therapy significantly inhibited Hela-O3 invasion compared to control, at MOI 3, 12, and 50 (Fig. 2C). Furthermore, *EDNRB* gene therapy inhibited Hela-O3 invasion in a dose dependent manner, with higher MOI producing a stronger inhibitory effect on invasion. *EDNRB* transduction at MOI 50 produced the strongest inhibitory effect on invasion without significant cellular death (determined through preliminary experiments at higher MOI doses). Finally, we tested the combination of *EDNRB* transduction at MOI 50 and macitentan at 4.5 µM. We showed that combination treatment produced a stronger inhibitory effect on invasion than control treatment or *EDNRB* gene therapy alone (Fig. 2D).

### Combination treatment of macitentan and *EDNRB* gene therapy inhibits oral cancer metastasis to cervical lymph nodes

**Figure 3:**
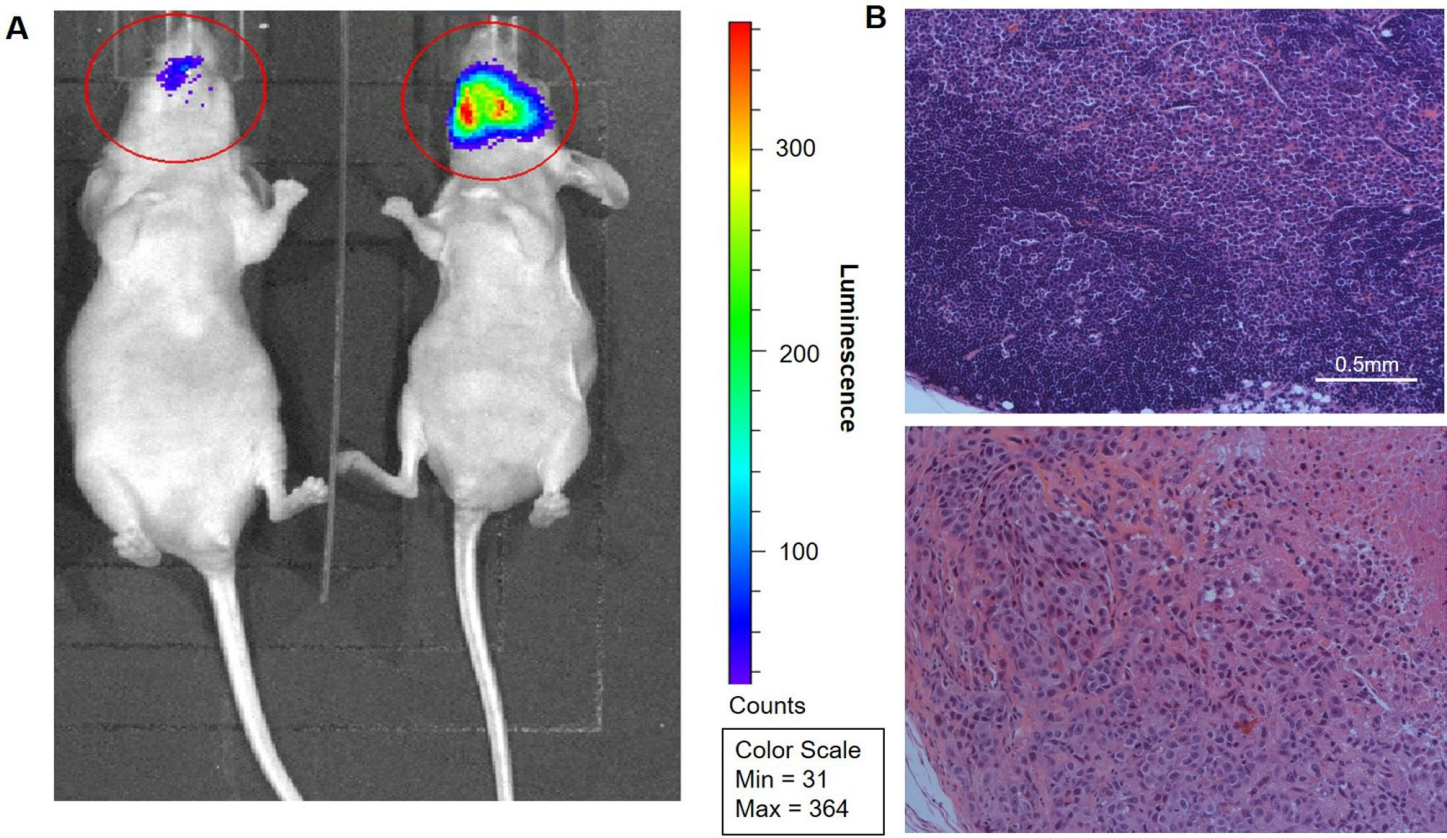
Representative images showing differences in metastasis patterns between treatment groups. (**A**) Bioluminescence images obtained using an Xenogen IVIS Lumina II bioanalyzer allows for tumor volume quantification and identification of cervical metastasis, with two representative images shown here: a mouse with tongue SCC without metastasis (left) and one with tongue SCC with cervical metastasis (right). The total luminescence signal is calculated for each region of interest (shown as red circles); the total signal is lower in the mouse on the left than the one on the right (9.47 × 10^3^ vs 8.37 × 10^4^). (**B**) Bilateral neck dissection specimens processed and stained with hematoxylin and eosin (H&E), with images shown at 10x, demonstrate normal lymph node architecture for the mouse with no cervical metastasis on bioluminescence imaging, and metastasizing carcinoma cells and central necrosis of the lymph node for the mouse with cervical metastasis.

We next determined whether combination treatment with *EDNRB* gene therapy and macitentan would inhibit neck metastasis in a mouse tongue SCC model established using the Hela-O3 cell line. Mice were monitored for weight loss according to our animal protocol; none of the mice in any of the experimental groups had significant weight loss (i.e., more than 15%) throughout the experimental time course. Imaging of the tongue cancer and cervical nodes were performed at 21 days using the Xenogen IVIS Lumina II bioanalyzer (Fig. 3A). Cervical node metastasis was quantified for each of the treatment groups, with three sections reviewed per mouse. For all animals where cervical node metastasis was present in one section, we also saw the presence of metastasis in the additional two analyzed sections (Fig. 3B). The rate of cervical metastasis was 46% for the control Ad-GFP/vehicle group (n = 13), 21% for the Ad-GFP/macitentan group (n = 14), 29% for the Ad-EDRNB/vehicle group (n = 14), and 0% for the Ad-EDNRB/macitentan group (n = 14). The combination treatment group (Ad-EDNRB/macitentan) had a significantly lower metastasis rate than the control group (Ad-GFP/vehicle) with *p* = 0.006. The groups treated with either *EDNRB* gene transduction or macitentan alone were not significantly different from the control group (Table 1).

**Table 1:** Frequency of neck metastasis in mouse treatment groups.

Treatment group	% Neck metastasis	*p* value (Fisher's exact, compared to Group 1)
1. Ad-GFP/Vehicle (n = 13)	0.46	
2. Ad-GFP/Macitentan (n = 14)	0.21	0.236
3. Ad-EDNRB/Vehicle (n = 14)	0.29	0.44
4. Ad-EDNRB/Macitentan (n = 14)	0.00	0.006

### Combination treatment of macitentan and *EDNRB* gene therapy does not affect oral cancer proliferation in vitro or in vivo

**Figure 4:**
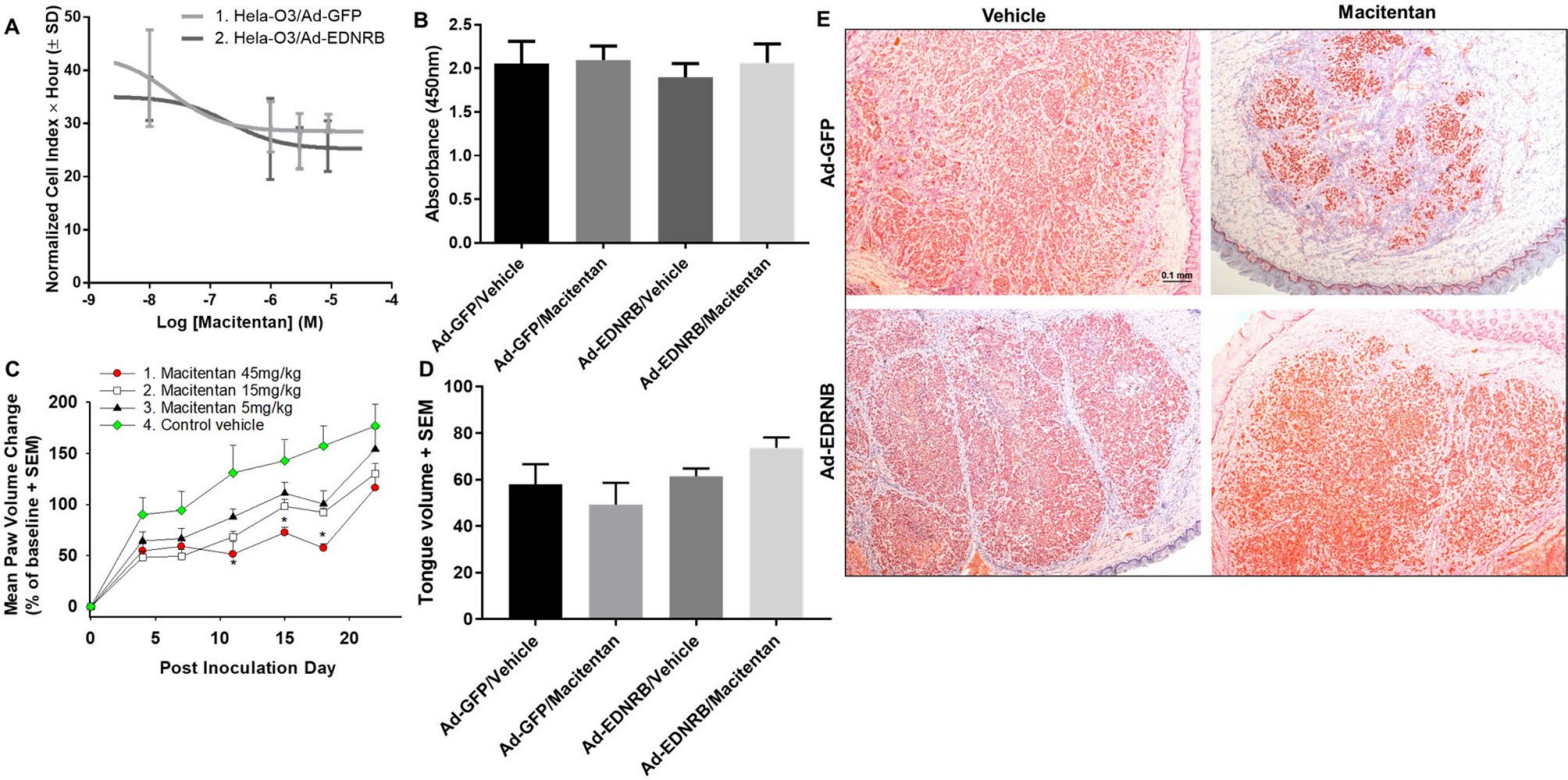
Macitentan treatment and *EDNRB* re-expression have no significant effect on proliferation in vitro or in a mouse model*.* (**A**) The two graphs show macitentan concentration vs. area under the curve (AUC) for Ad-GFP and Ad-EDNRB groups in a 72 h time course RTCA experiment, with AUC of hours 45 to 64 vs. log10 of macitentan concentration shown. *EDNRB* re-expression on Hela-O3 cells fail to produce a significant effect on proliferation compared to control Hela-O3/GFP. (**B**) Results of the MTS assay where absorbance is quantified as the index of proliferation demonstrates no difference in absorbance between the treatment groups. Ad-EDNRB transduction and macitentan treatment at IC50 dose fail to produce a significant anti-proliferative effect on Hela-O3 cells. (**C**) The graph shows change in paw volume from baseline. While the macitentan treatment at the highest dose (45 mg/kg) produces an antiproliferative effect between PID 11 and PID18 in a paw HSC-3 cancer model, this effect is not sustained (n = 8 female mice per treatment; *p < .05, one-way ANOVA, Holm Sidak test). When the entire experimental time course of each treatment group is compared using two-way RM ANOVA, there is a significant difference between the macitentan 15 mg/kg and 45 mg/kg treatment compared to the control group (see Table 2 for statistical summary), however when each time point is analyzed separately macitentan treatment does not have a sustained antiproliferative effect. (**D**) Combination Ad-EDNRB transduction and macitentan treatment in a tongue Hela-O3 cancer fail to produce a significant anti-proliferative effect compared to control treatment (black bar, *GFP* gene therapy and vehicle). (**E**) Ki-67 chromogenic staining (seen in these photomicrographs as a red stain) performed on mouse tongue carcinoma to evaluate for proliferation demonstrates significant proliferation in all treatment groups, indicating that treatment with macitentan and Ad-EDNRB does not significantly change carcinoma proliferation. The scale bar represents 0.1 mm.

We next focused on proliferation. We had shown that *EDNRB* gene therapy alone did not produce an anti-proliferative effect in oral SCC in vitro or in vivo^1^. Here we determined whether combination *EDNRB* gene therapy and macitentan would inhibit proliferation in vitro. Hela-O3 cells were transduced with either *EDNRB* or *GFP,* then treated with macitentan. The IC50s of macitentan was 2.30 × 10^–8^ M for Hela-O3-*GFP* cells, and 2.18 × 10^–8^ M for Hela-O3-*EDNRB* cells (Fig. 4A), which were not significantly different, suggesting that combination treatment did not produce a significant anti-proliferative effect. The MTS assay was used as an additional proliferative assay, and also showed no difference in proliferation between the control group, *EDNRB* gene therapy only, macitentan only, and the combination treatment group (Fig. 4B).

We used the paw cancer model established using HSC-3 cells to test the effect of macitentan treatment alone on cancer growth. The paw model provided two advantages over the tongue model: 1) the HSC-3 paw model was used in our previous study to test the effect of *EDNRB* re-expression on proliferation, so our results were directly comparable to the those of the previous study; 2) the paw volume could be measured throughout the experiment to track cancer growth, whereas repeated measurements were not possible with the tongue cancer model without sedating the mouse. The three tested doses (5 mg/kg, 15 mg/kg, and 45 mg/kg) were determined based on previous studies on mouse cancer models^4–6^. There was no significant weight loss, dehydration or lethargy in the vehicle or treatment groups. Macitentan treatment failed to inhibit cancer growth. The macitentan 5 mg/kg and 15 mg/kg groups showed no significant difference in cancer growth with the vehicle group throughout the experimental course. While there was a temporary inhibitory effect on cancer growth in the macitentan 45 mg/kg group between post inoculation days (PID) 11 and 18, this effect was not sustained at the end of the experiment on PID 22. By PID 22, there was no significant difference in paw volume change (i.e., cancer growth) between the vehicle group and three macitentan groups (Fig. 4C).

We next determined whether the combination of macitentan 45 mg/kg and *EDNRB* re-expression could inhibit cancer growth. Using the same paw model, we tested macitentan 45 mg/kg alone, *EDNRB* gene therapy alone, and the combination of the two treatments compared to control vehicle. By PID 28, the average paw volumes were as follows: Ad-EDNRB alone 0.28 ml, Ad-EDNRB/macitentan 0.39 ml, macitentan alone 0.37 ml, control vehicle 0.37 ml. There was no significant difference between the four treatment groups. The treatments were also tested in a mouse tongue SCC model established with Hela-O3. We inoculated Hela-O3 cells into the right lateral tongue. We monitored the mice in all groups for weight loss, dehydration and lethargy. On PID 21 we harvested tongue tissue and measured the volume of carcinoma. The average tumor volume for the four groups was: 58 ± 8.68 (SD) mm^3^ (Ad-GFP/vehicle, n = 8), 49.29 ± 9.39 mm^3^ (Ad-GFP/macitentan, n = 8), 61.5 ± 9.49 mm^3^ (Ad-EDNRB/vehicle, n = 8), 73.75 ± 12.75 mm^3^ (Ad-EDNRB/macitentan, n = 8), with no significant difference among the groups (Fig. 4D). Therefore, treatment with macitentan and *EDNRB* re-expression had no effect on cancer growth in the mouse tongue cancer model.

We performed Ki67 chromogenic staining to determine whether macitentan and *EDNRB* re-expression had an antiproliferative effect at the cellular level in the tongue cancer mouse model. We stained representative sections of the tongue cancers in each of the treatment groups. Ki67 staining was positive in all four treatment groups, indicating that macitentan and *EDNRB* re-expression did not significantly inhibit cancer proliferation (Fig. 4E). The slides were analyzed with Image J and mean values from the quantification were as follows: GFP/Vehicle 184.9, GFP/Macitentan 207.2, EDNRB/Vehicle 194.1, EDNRB/Macitentan 177.9, with no significant difference between the quantified results.

### Macitentan has an antinociceptive effect in an HSC-3 paw cancer model

**Figure 5:**
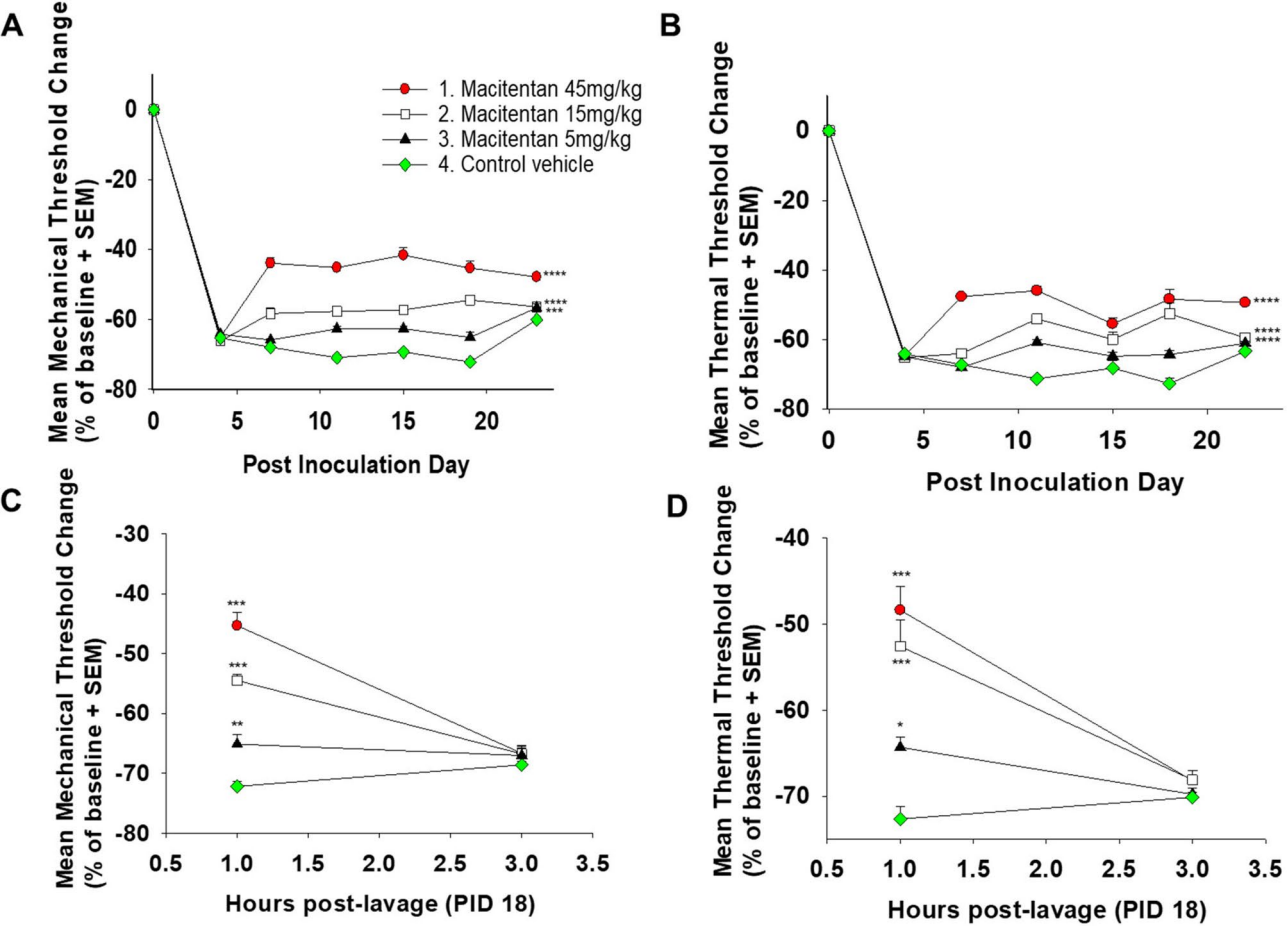
Macitentan has an antinociceptive effect in a paw HSC-3 mouse model. (**A**) and (**B**) HSC-3 inoculation into the hind paw to create paw tumors results in significant nociception represented by a decrease in both thermal and mechanical thresholds. Macitentan treatment at all three doses (5, 15, and 45 mg/kg) produces antinociception to both thermal and mechanical stimuli (n = 8 female mice per treatment, ***p < .001, ****p < .0001, two-way RM ANOVA, Holm Sidak test, see Table 2 for statistical summary). (**C**) and (**D**) The duration of macitentan’s antinociceptive effect (administered by oral gavage) is less than three hours. Macitentan at all three doses produces both thermal and mechanical antinociception at one hour, but not three hours, after administration (n = 8 female mice per treatment, *p < .05, **p < .01, ***p < .001, one-way ANOVA, Holm Sidak test).

We determined the antinociceptive potential of macitentan. Our previous work in patients and preclinical models established the endothelin pathway as a mediator of oral cancer pain, namely through secretion of high levels of ET-1 by cancer cells^1,7^. HSC-3 inoculation into the hind paw to produced significant thermal and mechanical nociception (Fig. 5), represented by a decrease in thermal and mechanical withdrawal thresholds in the control vehicle group following cancer inoculation. Macitentan treatment at all three doses (5 mg/kg, 15 mg/kg, and 45 mg/kg) produced an antinociceptive effect to thermal and mechanical stimuli (Fig. 5). The antinociceptive effect of macitentan was independent of its anti-tumor effect, since the three doses failed to produce an anti-tumor effect despite showing an anti-nociceptive effect.

To determine the duration of the antinociceptive effect produced by macitentan, we performed a time course experiment where we repeatedly measured thermal and mechanical withdrawal thresholds after macitentan treatment. The time course experiment was performed on PID 18 at one and three hours after macitentan administration. We determined that while macitentan produced an antinociceptive effect at all three administered doses (5, 15 and 45 mg/kg) at one hour after administration, this antinociceptive effect was not sustained after three hours (Fig. 5).

### Combination treatment of macitentan and *EDNRB* gene therapy produces antinociception in a Hela-O3 paw cancer mouse model

**Figure 6:**
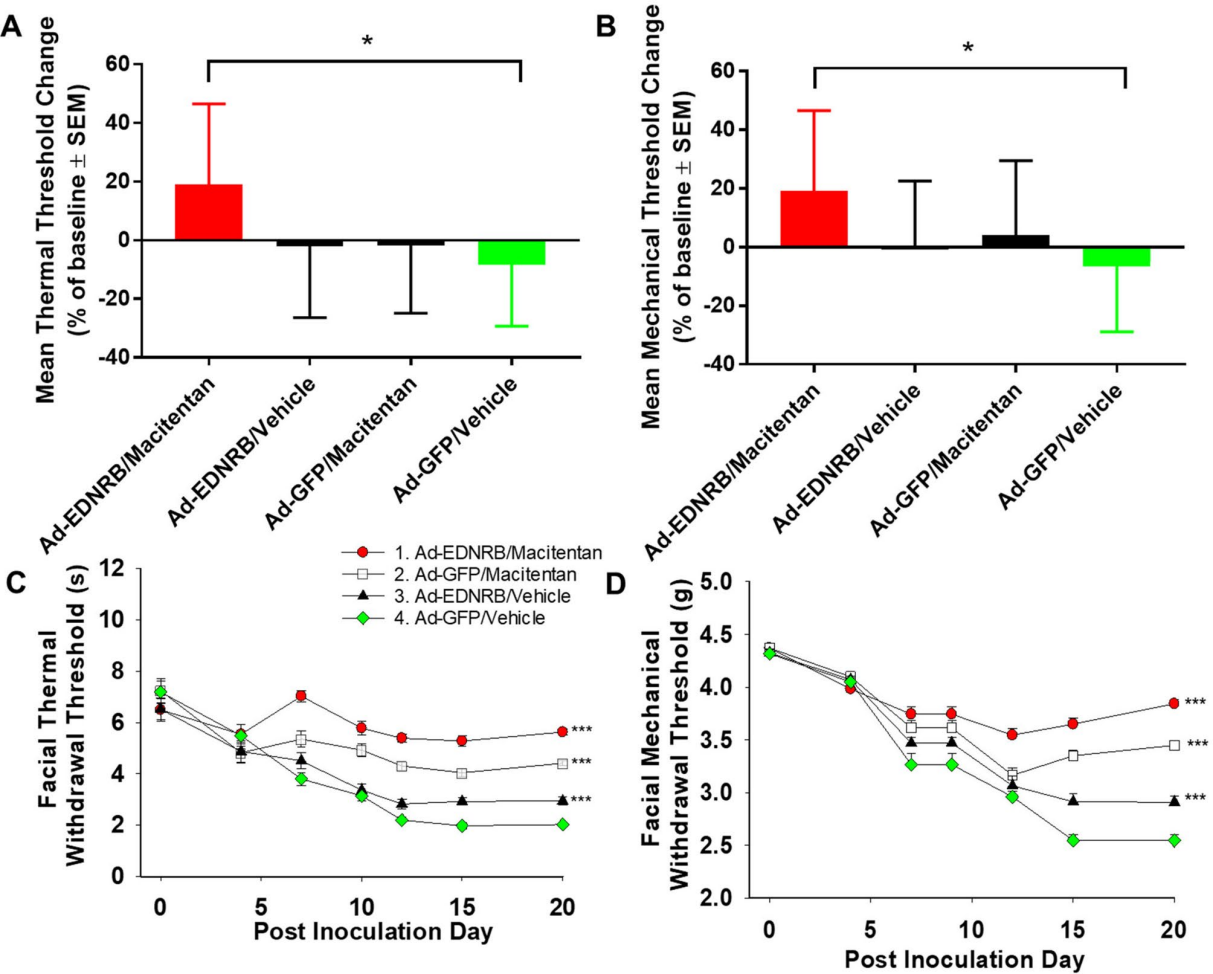
Combination treatment of macitentan and *EDNRB* gene therapy produces a significant antinociceptive effect to thermal and mechanical stimuli in a paw Hela-O3 cancer model and tongue Hela-O3 cancer model. (**A**) Graphs of thermal and (**B**) mechanical threshold change on PID 18 compared to baseline (with a negative change indicating increased nociception) demonstrate that combination treatment with macitentan and *EDNRB* gene therapy results in significant antinociception to thermal and mechanical stimuli compared to control vehicle in the Hela-O3 paw model (n = 8 female mice per treatment, *p < .05, one-way ANOVA, Holm Sidak test). (**C**) Graphs of thermal and (**D**) mechanical threshold change from baseline (day 0). While all three treatments produced antinociception compared to control vehicle (Ad-GFP/vehicle), the combination treatment of macitentan and *EDNRB* gene therapy produced the highest level of antinociception to both thermal and mechanical stimuli (n = 8 female mice per treatment, ***p < .001 two-way RM ANOVA, Holm Sidak test, see Table 2 for statistical summary).

We next determined whether the combination of macitentan and *EDNRB* gene therapy produced a more significant antinociceptive effect than either treatment alone. In this set of experiments we used a paw cancer model with HSC-3 cells inoculated into the hind paw. We determined the thermal and mechanical withdrawal thresholds at baseline prior to cancer inoculation; on PID 18 we measured thermal and mechanical hypersensitivity in each of the treatment groups. The mean threshold reduction (i.e., level of nociception) was highest in the control vehicle group. The Hela-O3 mouse model did not produce as much nociception as the HSC-3 paw model (Fig. 6; control vehicle group had a > 60% reduction from baseline for both mechanical and thermal thresholds). Of the three treatment groups, only the combination of macitentan and *EDNRB* gene therapy group had significantly higher thermal and mechanical thresholds than the control vehicle group. The mean threshold change for the monotherapy macitentan and *EDNRB* gene therapy groups were lower than the control group, but did not reach statistical significance (Fig. 6).

### Combination treatment of macitentan and *EDNRB* gene therapy produces antinociception in a Hela-O3 tongue cancer mouse model

We assessed facial mechanical and thermal hypersensitivity^8^ in mice with tongue cancer created from Hela-O3 inoculation. Combination treatment with macitentan and *EDNRB* gene therapy produced the highest antinociceptive effect of all the treatments, although macitentan or *EDNRB* gene therapy alone also produced an antinociceptive effect to mechanical and thermal stimuli (Fig. 6; Table 2).

**Table 2:** Statistical summary.

	Two-way RM ANOVA				Holm-Sidak post hoc	
	Effects	DF	F	*p*	Groups	*p*
Figure 4C	Treatment	3	6.897	0.0039	1 vs 4	0.0039
	Time	6	122.4	0.0001	2 vs 4	0.0182
	Interaction	18	2.16	0.0093	3 vs 4	0.1283
Figure 5A	Treatment	3	221.1	< 0.0001	1 vs 4	< 0.0001
	Time	6	1193	< 0.0001	2 vs 4	< 0.0001
	Interaction	18	17.53	< 0.0001	3 vs 4	< 0.0001
Figure 5B	Treatment	3	170.7	< 0.0001	1 vs 4	< 0.0001
	Time	6	1784	< 0.0001	2 vs 4	< 0.0001
	Interaction	18	31.14	< 0.0001	3 vs 4	0.0002
Figure 6C	Treatment	3	70.03	< 0.0001	1 vs 2	< 0.0001
	Time	6	49.15	< 0.0001	1 vs 3	< 0.0001
	Interaction	18	7.007	< 0.0001	1 vs 4	< 0.0001
					2 vs 3	< 0.0001
					2 vs 4	< 0.0001
					3 vs 4	0.1448
Figure 6D	Treatment	3	70.03	< 0.0001	1 vs 2	0.0073
	Time	6	49.15	< 0.0001	1 vs 3	< 0.0001
	Interaction	18	7.007	< 0.0001	1 vs 4	< 0.0001
					2 vs 3	< 0.0001
					2 vs 4	< 0.0001
					3 vs 4	0.0142

### Macitentan inhibits trigeminal neuron activation in vitro

**Figure 7:**
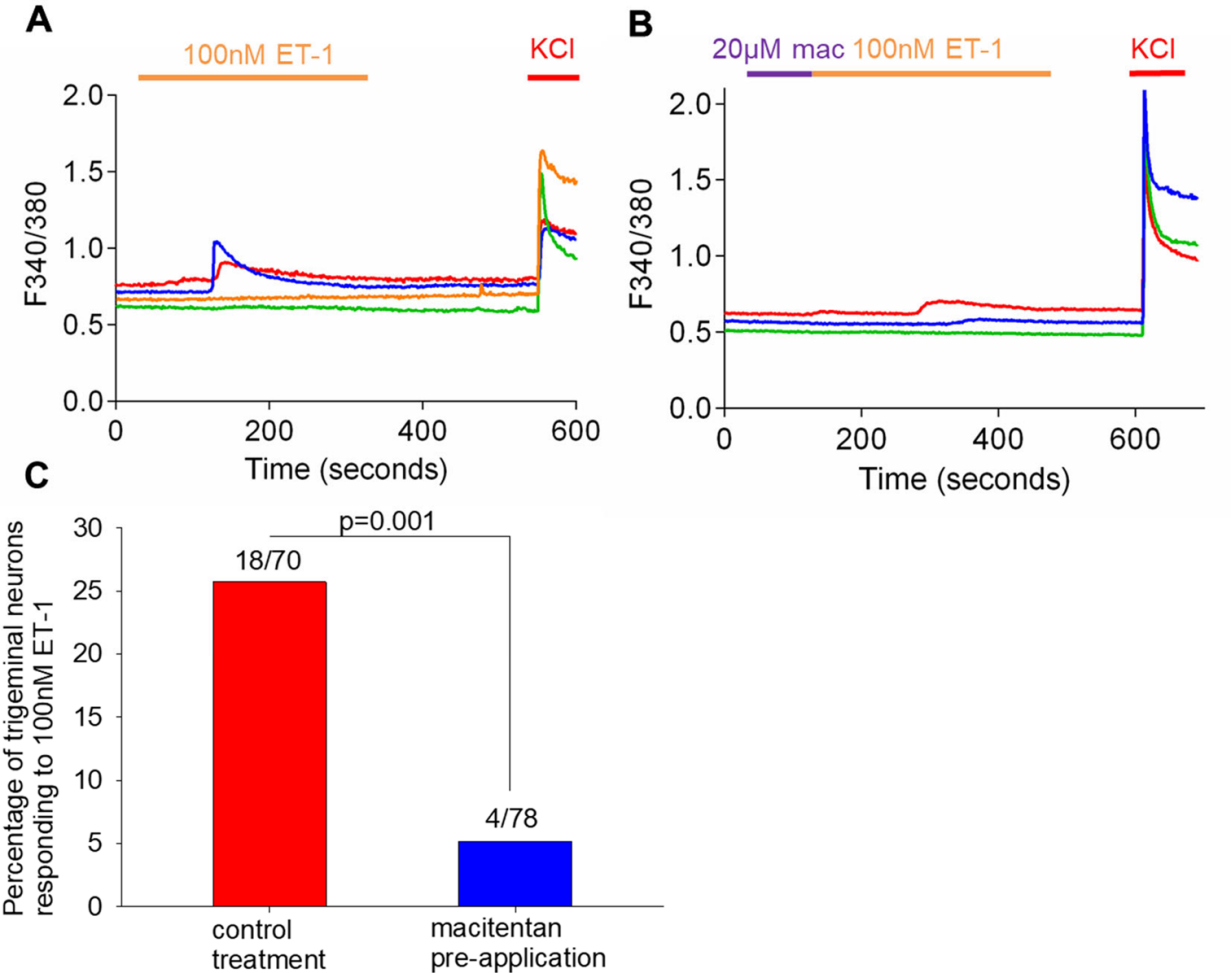
Macitentan significantly inhibits trigeminal neuron activation to endothelin-1. (**A**) We perform ratiometric calcium imaging and determine that 18/70 (26%) of neurons in a population of dissociated mouse trigeminal neurons are activated by ET-1 (at a dose of 100 nM). This percentage is similar to previously reported percentages in rat trigeminal neurons. (**B**) and (**C**) We then pre-treat the dissociated trigeminal neurons with 20 µM macitentan and show that pre-treatment significantly reduces the number of trigeminal neurons that are activated by ET-1 (*p* = 0.001).

We used real time calcium imaging to determine whether macitentan produced antinociception through inhibition of calcium-dependent neuronal activation. We initially quantified changes in calcium levels in neurons in response to ET-1. We treated the neurons with 100 nM ET-1. To choose the neurons that were alive in our analyzed fields we treated the neurons with KCl as live neurons respond to KCl with a large calcium influx. Of those with a positive KCl response we determined the change in the 340/380 nm ratio during ET-1 treatment, using 0.2 ratiometric change as a cutoff for a positive response^9^. We found that 18 of 70 analyzed neurons (26%) had a positive calcium response to ET-1 (Fig. 7). This fraction of ET-1 responsive mouse trigeminal neurons was comparable to results in rat trigeminal neurons, where 29% of neurons were responsive to ET-1^10^. We then pretreated the neurons with 20 µM macitentan prior to ET-1 application. We found that macitentan pretreatment significantly inhibited calcium response (i.e., neuronal activation) to ET-1, with 4/78 (5%) of analyzed neurons responding to ET-1. This inhibitory effect was statistically significant (*p* = 0.001).

## Discussion

### Endothelin axis, cancer invasion and metastasis

This study determines the role of the endothelin axis on oral cancer metastasis, proliferation and pain. Endothelin axis proteins have been detected in different malignancies, including ovarian, breast, melanoma, colorectal, bladder, and oral SCC^11^. ET-1 mediates metastasis through paracrine regulation of tumor-stromal interactions^12^. ET-1 secreted by cancer cells induce macrophage chemotaxis, vascular smooth muscle proliferation and angiogenesis, which result in cancer cell migration and metastasis^12,13^. The endothelin axis contributes to metastasis in other cancers through ET-1 activation of ET_A_R^12^. Our previous work shows that oral SCC cells secrete higher levels of ET-1 than other cancers, and ET-1 levels are higher in saliva of oral SCC patients than normal subjects^7^. In oral SCC, ET-1 activates the epidermal growth factor receptor (EGFR) to induce cell motility, which is crucial to metastasis^14^. On the other hand, ET_A_R antagonists, which inhibit ET-1 activation of this receptor, attenuate growth and invasion of oral SCC cells^15^. Endothelin pathway antagonists have been used in phase II and III clinical trials of metastatic prostate cancer without significant effect. However these trials use ET_A_R-specific antagonists^16^ without addressing the contribution of ET_B_R. The role of ET_B_R in metastasis across different cancer subtypes is poorly understood, and the lack of treatment benefit with ET_A_R antagonists in clinical trials points to the need to shift the focus to ET_B_R. We have shown that epigenetic silencing of *EDNRB*, the gene for ET_B_R, correlates with neck metastasis in oral SCC patients^1^. Our finding that ET_B_R gene silencing increases the likelihood of oral SCC metastasis suggests that the two ET receptors have dichotomous functions, and need to be differentially targeted. Our treatment strategy in this study, which exploits the distinct functions of the two receptors, through concurrent ET_A_R inhibition and ET_B_R re-expression, effectively inhibits oral SCC metastasis in vitro and in a preclinical model. While the combination treatment of macitentan and *EDNRB* gene therapy do not impact cancer proliferation, this negative result does not discount the endothelin axis as an important target for future studies on oral cancer treatment, because metastasis, not proliferation, is the single most important predictor of survival in oral SCC patients^2,17^.

### Endothelin axis and cancer pain

ET-1 has a dichotomous effect with regard to pain. ET-1 activation of its receptors can produce both pain and analgesia; the response depends on which receptor, and which cell, is activated^18,19^. *EDNRB* is significantly hypermethylated in cancer tissue of oral SCC patients compared to their contralateral normal tissue; over-expression and activation of ET_B_R (by ET-1 in the cancer microenvironment) reduces cancer pain via the release of β-endorphin^1^. Our findings over the last decade have led us to our approach in this study to further define how the endothelin axis contributes to oral SCC pain. Concurrent ET_A_R inhibition with macitentan and *EDNRB* re-expression produces a significant antinociceptive effect in two separate mouse cancer models. At the level of the neuron, macitentan dampens calcium influx in neurons in response to ET-1. Our preclinical findings set the stage for a future clinical trial to evaluate the analgesic potential of macitentan in cancer patients. Cancer pain remains poorly treated, with opioids being the only effective analgesic, putting cancer patients at risk of opioid dependence. There is a pressing need for alternative analgesics, especially with the current opioid epidemic and its devastating effects on patients and the healthcare system. Ineffective cancer pain treatment not only erodes quality of life, it decreases survival in cancer patients^20^. Oral cancer patients in particular are at high risk of poor pain control and opioid dependence^21,22^.

### Dimeric binding of ET_A_R and ET_B_R and binding affinities of ET_A_R and ET_B_R for ET-1

ET_A_R and ET_B_R associate as heterodimers via binding to the bivalent ET-1 ligand. An ET_A_R antagonist disrupts the heterodimer and liberates ET_B_R to bind ET-1 with a nine-fold greater affinity than either the ET_A_R or the A-B dimer^23^. Thus, an appropriate ET_A_R antagonist could free ET_B_R to bind ET-1 in the cancer microenvironment, which results in endogenous opioid secretion—a potential approach to control cancer pain at its origin. Previously developed endothelin receptor antagonists include atrasentan and bosentan. Macitentan, unlike bosentan and ambrisentan, is an insurmountable antagonist with low receptor dissociation rates across a wide range of ET-1 concentrations^24^. In addition to its high affinity for ET_A_R, macitentan has a low affinity for ET_B_R, pharmacology that is critical for ET_B_R activation by ET-1. Macitentan could be administered orally and has already been used in clinical trials to treat pulmonary hypertension^25^. In addition to establishing the anti-cancer effects of macitentan on oral SCC, we define a new role for macitentan as an analgesic to treat cancer pain. Our treatment strategy involving macitentan with *EDNRB* gene therapy more effectively inhibits oral SCC metastasis and pain than macitentan alone. While directly translating this treatment strategy to clinical trials is a complex task, the translational potential of our study lies in demonstrating a precision medicine approach to treating oral cancer metastasis and pain. We identify a dysregulated pathway in the cancer tissues of oral cancer patients, design therapy to correct dysregulation of component genes within the pathway using a readily available drug and gene therapy, and effectively treat metastasis and pain in a preclinical model. Furthermore we highlight the importance of leveraging the dichotomous roles of the ET_A_R and ET_B_R receptors, which has largely been ignored in preclinical and clinical studies across different cancer subtypes^16^. Our findings highlight the endothelin axis as an important target not only for cancer metastasis, but also for cancer pain, a problem that remains poorly treated despite advances in cancer treatment leading to improved survival.

## Methods

### Cell culture

*Cancer cells:* The human oral squamous cell carcinoma cell lines, HSC-3 and Hela-O3, were cultured in Dulbecco’s Modification of Eagle’s Medium (DMEM) with 4.5 g/L glucose, l-glutamine and sodium pyruvate, supplemented with 10% fetal bovine serum (FBS), and cultured at 37 °C in 5% CO_2_. HSC-3 was purchased from ATCC and used fewer than 6 months after resuscitation. Hela-O3 was obtained from Dr. Roberto Weigert at the National Cancer Institute and tested for Mycoplasma by PCR (ATCC) prior to use.

*Neurons:* Mouse trigeminal ganglia were harvested and cultured as previously described^26^. Trigeminal ganglia were isolated, transferred into Hank’s Balanced Salt Solution (HBSS) and enzyme-digested by incubation with papain (Worthington), collagenase type II (Worthington), and dispase type II (MB). Dissociated neurons were plated on glass coverslips coated with poly-d-lysine and laminin and maintained at 37 °C at 5% CO_2_/95% air in F12 media (Life Technologies) with 5% FBS.

### Transfection of luciferase

The Hela-O3 cell line was transfected with a plasmid encoding the luciferase gene to allow for live animal tumor imaging. Plasmid DNA, encoding for luciferase (gWIZ luciferase) under the control of the cytomegalovirus promoter/enhancer was obtained from Genlantics (San Diego, CA). We used a modified transfection technique with a nonviral hybrid vector, a HIV-1 Tat peptide sequence modified with histidine and cysteine residues combined with a cationic lipid^27^.

### Transduction of *EDNRB*

Human cDNA of *EDNRB* containing a C-terminal GFP tag (OriGene) was subcloned into a shuttle plasmid. Subcloning and viral particle purification were completed through Viraquest. Oral SCC cells (HSC-3 or Hela-O3) were transduced with recombinant adenovirus (Ad-EDNRB or Ad-GFP) at increasing multiplicities of infection (MOI) to determine transduction efficiency. Transduction was performed in DMEM with 2% fetal bovine serum (FBS) and the aforementioned supplements^1^.

### In vitro migration and invasion assay

The BD Biosciences invasion assay was used according to manufacturer’s recommendations. The assay consisted of an upper invasion chamber insert that fit into a 24-well cell culture plate. The upper invasion chamber was coated with BD Matrigel matrix to allow for assessment of invasive capacity. For each treatment, non-coated invasion chambers were used as the control. Equal numbers of Hela-O3 cells (2 × 10^5^) in DMEM supplemented with 0.4% FBS were added to each upper chamber. The cells had either been transduced with *GFP* or *EDNRB*^1^*.* Macitentan was dissolved in DMEM. The bottom wells in the 24-well culture plate were filled with DMEM supplemented with 5% FBS, which served as a chemoattractant. The upper invasion chambers were placed in the cell culture plate and incubated at 37 °C for 16 h. At the end of the incubation period, cells from the upper surface of the filter were wiped off with a cotton swab. The lower surface of the filter was stained with DiffQuik (Dade Behring, Switzerland). The number of cells that migrated to the bottom of the chamber were counted in the light microscope on ten randomly selected fields at 10 × magnification. Counting was performed by an investigator who was blinded to the treatment groups. The mean number of cells was calculated per field. Three sets of experiments were carried out, each in quadruplicate. The percent invasion was calculated for each well by dividing the mean number of cells in each well by the mean number of cells of all control wells for the given treatment.

### RTCA assay

The xCELLigence Real-time Cell Analyzer (ACEA) was used to quantify change in impedance and an index of cell proliferation. 10^3^ Hela-O3 cells, transduced with either Ad-GFP or Ad-EDNRB, were grown overnight in DMEM supplemented with 5% FBS to obtain a baseline. After a baseline was obtained, macitentan or vehicle control was added to each well. The cells were continuously monitored every 15 s for 72 h. The change in impedance compared to baseline was calculated and converted into a cell index by the Real-time Cell Analyzer software.

### MTS assay

The MTS assay was used to quantify the effect of macitentan treatment and *EDNRB* re-expression on Hela-O3 proliferation. 2 × 10^4^ Hela-O3 cells that were transduced with either *GFP* or *EDNRB* (at 12 MOI) were seeded in each well in a 96-well plate. Cells were seeded with either macitentan 4.5 µM or vehicle (DMSO) in 5% FBS supplemented DMEM. Each treatment group was seeded in 16 wells. Cells were incubated at 37ºC for 24 h. 20 μl of MTS (Promega BioSciences, San Luis Obispo, CA) was added to each well and incubated at 37 °C for 2 h. The absorbance of each well was read at 450 nm. The absorbance value was averaged for each of the four treatment groups.

### Calcium imaging

Dissociated neurons were seeded onto glass coverslips, loaded with 1 µM of the cell permeable calcium sensitive dye, Fura 2AM (Molecular Probes) for 30 min and washed with HBSS before use. Coverslips containing cells were placed in a chamber with constant infusion of HBSS at room temperature. Fluorescence was detected by a Nikon Eclipse TI microscope at 340 and 380 excitation wavelengths and analyzed with the TI Element Software (Nikon). HSC-3 cells were counted as responsive to 10 µM [D-Ala^2^, N-MePhe^4^, Gly-ol]-enkephalin (DAMGO) infusion and neurons were counted as responsive to cancer supernatant infusion if the 340/380 ratio was ≥ 0.2 from baseline^28^.

### Mouse cancer model

All mouse experiments were approved by the Institutional Animal Care and Use Committee (IACUC) at New York University and performed in accordance with IACUC regulations. A mouse paw cancer pain model was produced by inoculating HSC-3, an oral squamous cell carcinoma cell line, into the right hind paw of athymic BALB/c as previously described^7^. 24 h prior to inoculation, cancer cells were transduced with Ad-EDNRB or Ad-GFP. The mice were divided into two groups and inoculated in the right hind paw with the respective cell types: (1) Ad-EDNRB and (2) Ad-GFP. Paw volume measurements were performed with a plethysmometer (IITC Life Sciences) as described^1^ and used as an index of cancer growth. A mouse tongue cancer model was produced by inoculating Hela-O3 into the right tongue of athymic BALB/c mice. The Hela-O3 cell line (formerly OSCC3) metastasizes to the cervical nodes when injected into oral cavity of athymic mice^3^.

### Macitentan treatment

Macitentan, a dual type A and type B endothelin (ET) receptor antagonist, which was provided by Actelion, was dissolved in vehicle containing 0.5% methylcellulose aqueous solution and 0.05% Tween 80. The solution was administered orally according to body weight at 15 mg/kg, 30 mg/kg, or 45 mg/kg for dosing experiments, and at 45 mg/kg for all other experiments. For in vitro experiments macitentan was dissolved directly into cell media.

### Mechanical allodynia measurement

We determined mechanical allodynia using two separate cancer pain models, a tongue cancer model and a paw cancer model. We quantified facial allodynia using a graded series of von Frey filaments^29^. Testing was performed on day 0 prior to inoculation and twice a week after inoculation. The graded von Frey assay is a valid assay for quantifying allodynia in mouse models of orofacial cancer^30^.We quantified paw withdrawal thresholds using an electronic von Frey anesthesiometer as we have described^1^. Testing was performed on day 0 prior to inoculation and then twice a week after inoculation.

### Thermal hyperalgesia measurement

We determined thermal hyperalgesia using the facial cancer model and paw cancer models. Thermal hyperalgesia was assessed as previously described^31^ using a focused projection bulb to deliver a thermal stimulus to the right whisker pad or right footpad of each mouse with a cutoff of 20 s. Facial withdrawal or paw withdrawal to heat was calculated as a mean of six measurements. Testing was performed on day 0 prior to inoculation and then twice a week after inoculation, on the same day as mechanical allodynia testing.

### Bioluminescence imaging

Bioluminescence images were obtained using a Xenogen IVIS Lumina II bioanalyzer. Twenty minutes prior to imaging, the mice were injected with 200 μl d-luciferin (Caliper Life Science). They were then anesthetized with isoflurane gas and kept under anesthesia with 2% isoflurane. Mice were then placed in a prone position in the IVIS imaging system and one whole body scan was acquired. Light emission was collected and the intensity was represented as the number of photons/cm^2^ within a region of interest (ROI). Luciferase expression was quantified using Living Image Software version 4.0. For mice in all experimental groups the ROI was chosen as the facial region, to quantify the extent of cancer growth in the tongue.

### Tissue harvesting

Mouse tissues were harvested at the end of the experiment. For the mouse tongue cancer model, the tongue and lymph nodes of the neck were harvested, fixed in 4% paraformaldehyde and embedded in paraffin. Cancer volume of the tongue was calculated after tissue harvest by measurement of the length, width and depth of the cancer. Three 5 µm sections were cut for hematoxylin and eosin staining. The lymph node sections were analyzed for evidence of cancer metastasis by a board-certified oral pathologist (Dr. King Chong Chan) who was blinded to the treatment groups. Cervical node metastasis was marked as absent if all three sections did not demonstrate presence of cancer cells. Cervical node metastasis was marked as present if at least one of the three sections demonstrated presence of cancer cells.

### Patient tissue array analysis

The study was approved by the Institutional Review Board at New York University and carried out in accordance with relevant guidelines and regulations. Informed consent was obtained from all patients, with eligible patients including those 18 years of age or over with biopsy-proven oral cavity squamous cell carcinoma, and no previous history of head and neck cancer treatment. Snap frozen tissue of the cancer and contralateral normal tissue were taken at the time of ablative surgery from 22 patients with oral cancer. Disease subsites included tongue, maxillary and mandibular gingiva, and floor of mouth. RNA and DNA were extracted according to Qiagen AllPrep protocol (Qiagen). RNA and DNA were processed to prepare for the Illumina 450K Methylation Array and Gene Expression Array. For each of the endothelin pathway genes that were known to be expressed in cancer cells (i.e., *ECE1, ECE2, EDN1, EDNRA, EDNRB*) we generated a correlation matrix of gene expression and DNA methylation. We then fit a linear regression model for each CpG site and each transcript of a given gene.

### Statistical analysis

Results were analyzed using Sigma Plot version 13.0 or GraphPad Prism version 7.01.
